# State Wellness Policy Requirement Laws Matter for District Wellness Policy Comprehensiveness and Wellness Policy Implementation in the United States

**DOI:** 10.3390/nu13010188

**Published:** 2021-01-09

**Authors:** Jamie F. Chriqui, Julien Leider, Lindsey Turner, Elizabeth Piekarz-Porter, Marlene B. Schwartz

**Affiliations:** 1 Division of Health Policy and Administration, School of Public Health, University of Illinois Chicago, Chicago, IL 60612, USA; lindseyturner1@boisestate.edu; 2 Institute for Health Research and Policy, University of Illinois Chicago, Chicago, IL 60608, USA; jleide2@uic.edu; 3 College of Education, Boise State University, 1910 University Drive, Boise, ID 83725, USA; epiekarz@uic.edu; 4 Rudd Center for Food Policy and Obesity, Department of Human Development and Family Sciences, University of Connecticut, 1 Constitution Plaza, Hartford, CT 06103, USA; marlene.schwartz@uconn.edu

**Keywords:** wellness policy, legal epidemiology, policy implementation, school nutrition, school physical activity

## Abstract

Beginning with the school year 2006–2007, U.S. school districts participating in the federal Child Nutrition Programs were required to adopt and implement a local wellness policy (LWP) that included goals and/or standards for nutrition education, school meals, other foods sold or served in schools, and physical activity. A primary challenge with LWPs has been inconsistent implementation. This study examined whether state wellness policy requirement laws and district LWP comprehensiveness influence district level implementation, using law/policy data from the National Wellness Policy Study and school food authority (SFA)-reported district LWP implementation from the School Nutrition and Meal Cost Study. Generalized linear and structural equation models were used, controlling for SFA and district characteristics. SFAs in states with wellness policy requirement laws (vs. those in states without) reported implementing significantly more practices (59.56% vs. 44.57%, *p* < 0.01). State wellness policy requirement laws were associated with district LWP comprehensiveness (coeff.: 0.463; 95% CI: 0.123, 0.803) and district-level implementation (coeff.: 1.392; 95% CI: 0.299, 2.485). District LWP comprehensiveness was associated with district implementation (coeff.: 0.562; 95% CI: 0.072, 1.053), but did not mediate the state law–district implementation relationship. This study highlights the important role that state laws and district LWPs can play in facilitating wellness policy implementation.

## 1. Introduction

Beginning with school year 2006–2007, all local education agencies (LEAs; school districts) in the United States that participated in the federal Child Nutrition Programs (including the National School Lunch Program and/or the School Breakfast Program) were required to adopt and implement a local wellness policy (LWP) [1]. Originally, LWPs were required to include goals for nutrition education, physical activity, and other school-based activities that promote student wellness; an assurance that school meal nutrition guidelines meet the minimum federal school meal standards; guidelines for foods and beverages sold or served outside of the school meal programs; and implementation plans [1]. The Healthy, Hunger-Free Kids Act of 2010 renewed and expanded the LWP requirements, including additional reporting and evaluation provisions and a requirement to designate one or more district officials to ensure that schools comply with the LWP [2]. The Act also gave the U.S. Department of Agriculture (USDA) the authority to issue regulations governing LWP content and implementation, which the agency promulgated in July 2016 [3]. Concurrent with the LWP final rule, the USDA also updated and expanded its regulations governing each state agency’s administrative review process for local implementation of the National School Lunch Program and the School Breakfast Program to include state agency oversight regarding LWP adoption and implementation [4].

Since the original LWP mandate, there has been a patchwork of district LWPs and state laws governing the school wellness, health, nutrition, and physical activity environments in the United States [5,6,7,8]. There are tools for measuring the comprehensiveness (scope) and strength (required provisions) of LWPs [9,10,11] and concomitant state laws [11,12,13,14]. Numerous studies within individual states and nationwide have examined LWP content, consistently demonstrating that while LWPs are comprehensive (i.e., addressing many topics), they are often weak (i.e., simply encouraging rather than requiring specific actions) [5,6,15,16,17,18,19,20,21,22]. Qualitative and descriptive studies have examined factors influencing the implementation of LWP [17,23,24,25,26,27,28,29,30,31]. Additionally, a number of studies have documented the association between LWPs or components of the LWP (e.g., physical activity provisions or school food provisions) and concomitant school practices [30,32,33,34,35,36,37,38,39,40,41,42,43,44,45,46,47,48,49,50,51,52] and/or student outcomes [19,53,54,55,56,57].

At the same time, there have been a plethora of studies examining the association or impact of state laws governing elements of LWPs (e.g., laws related to school food, nutrition, and physical activity and physical education environments) on district LWPs, school practices, and student outcomes [35,37,39,40,42,58,59,60,61,62,63,64,65,66,67,68,69,70,71,72]. In most instances, these LWP content-related state laws (e.g., school food requirements) were associated with school practices and student outcomes; however, these state laws were not mandated by federal law like the LWP. In addition to state laws governing topics covered in LWPs (e.g., nutrition education, school foods), 16 states and the District of Columbia have independently enacted a state wellness policy requirement law [i.e., laws that specifically require that school districts submit the LWP for review and/or compliance to the responsible state agency (typically the state education agency)] (unpublished data compiled for this study). The states’ potential role in this area is key because the federal government does not provide consistent and tailored support to districts for LWP implementation; thus, state agencies are where districts turn for guidance. States, through their legislative and regulatory powers, can reinforce, bolster, or complement the federal mandate and, with the updates to the USDA Administrative Review rule, state agencies now have oversight over LWPs as part of their compliance responsibilities [4].

The purpose of this study was to fill a gap in the literature and examine the potential role that states can play in providing a framework for LWPs by examining whether having a specific state wellness policy requirement law (i.e., state requirement that districts submit their LWP to the state for review and compliance) is associated with district LWP comprehensiveness (i.e., breadth of content) and LWP implementation nationwide. We hypothesized that districts in states with a wellness policy requirement law would have more comprehensive LWPs (address more LWP topics) and have higher rates of implementation. We also hypothesized that LWP comprehensiveness would mediate the relationship between state wellness policy requirement laws and rates of LWP implementation.

## 2. Materials and Methods

### 2.1. Data and Design

The School Nutrition and Meal Cost Study (SNMCS) was conducted by Mathematica Policy Research in the 2014–2015 school year for the United States Department of Agriculture, Food and Nutrition Service [73]. One aspect of the SNMCS data collection included a survey of School Food Authority (SFA) directors [74]. SFAs are designated by the USDA as the governing body responsible for school food service operations in a given district (or districts as some small districts operate together under a single SFA) [4,75]. Because LWPs are required of all LEAs participating in the federal school meal programs, and because SFAs are responsible for oversight over those programs and reporting to the given state agency on district and school compliance with the federal Child Nutrition Program standards [76], they play a pivotal role in helping LEAs to oversee LWP implementation and compliance. To this end, the SNMCS SFA Director Survey included a wide range of questions, including items related to reimbursable meals and competitive foods and LWP implementation. The current study utilized items related to LWP implementation described further below. Mathematica Policy Research linked the SNMCS SFA-reported LWP implementation data with state law and LWP data for the SNMCS districts compiled by the National Wellness Policy Study using district identifiers. A de-identified data set containing the SNMCS SFA Director Survey data along with the corresponding state law and LWP data was then provided to National Wellness Policy Study staff at the University of Illinois Chicago (UIC) for this analysis. The survey-weighted data are nationally representative of all public SFAs that offer the National School Lunch Program. This study was deemed to “not involve human subjects” by the UIC Institutional Review Board (protocol #2020-0448).

### 2.2. Measures

#### 2.2.1. SFA Director Survey Measures

SNMCS included 548 SFAs; 518 of them completed the SFA Director Survey. As part of the survey, directors who reported having a district wellness policy (99%) were asked to review a list of 11 potential and required wellness policy components aligned to the federal wellness policy requirements and to indicate for each one whether it was addressed in their district wellness policy, and if so, the extent to which it had been implemented. These components included: nutrition education; nutrition promotion; physical education (PE); daily physical activity (outside of PE); restrictions on the use of food or food coupons as student rewards; access to competitive foods during school hours; minimum amount of time for students to eat lunch; staff wellness program; plan for measuring implementation of the policy, including the extent in compliance with the policy; plan for describing the progress made towards attaining the goals of the policy; and plan for informing the public about the wellness policy content and implementation. For each component, SFA directors were asked to indicate if it was “addressed in policy and fully implemented”, “addressed in policy and partially implemented”, “still being planned”, or “not addressed in policy”. For this analysis, we created a scale with a potential range of 0–11 that represented the number of components that were fully implemented.

#### 2.2.2. State Wellness Policy Requirements and District LWP Data

The state wellness policy requirement laws and district LWP data were compiled through primary legal research by the National Wellness Policy Study [6,8]. These data reflect state laws and LWP in effect the day after Labor Day 2014 (2 September 2014), a proxy for the beginning of the school year. District policies were collected using established methods [6,77] via Internet research with electronic mail and telephone calls to district officials to verify the Internet collection and to obtain any missing policy information. State statutes and administrative regulations were collected using the LexisNexis and Westlaw subscription services [78,79].

At the state level, a dichotomous indicator was created to measure the presence or absence of a state wellness policy requirement law, which reflected whether state law required districts to submit their LWP for review and/or compliance to the state agency (1 = yes, 0 = no). At the district level, the LWPs were coded using the National Wellness Policy Study coding tool [11]; from the National Wellness Policy Study coded data, 12 items directly corresponded to the LWP implementation-related items captured in the SNMCS SFA Director Survey. These items were compiled into a LWP comprehensiveness scale, which measured the extent to which the following 12 items were addressed in the LWPs: goals for nutrition education; marketing healthy choices; goals for physical education; physical activity throughout the day; meeting Smart Snacks (computed based on coding of nutrition standards for vending machines, school stores, à la carte, and fundraisers); measuring implementation; reporting on progress in meeting wellness policy goals; reporting to the public; food as a reward/punishment; adequate time to eat; staff wellness programs; and recess frequency or amount (computed based on whether daily or less than daily recess frequency or amount was addressed; this was only coded for elementary school). The scale ranged from 0–100 and represented the percentage of these items that were addressed in the LWP. Policies were coded separately for their applicability at different school levels (elementary, middle, and high school levels); the mean value of this scale across grade levels was computed for analysis at the SFA level. The scale was then split into three tertiles with equal survey-weighted frequency, and this tertiled measure was used for analyses in order to better understand associations involving relatively more (vs. less) comprehensive LWPs.

#### 2.2.3. SFA and District Controls

Analyses controlled for SFA and district characteristics. The district child poverty rate and SFA size were included in the SNMCS data files and derived from the 2011 Census Bureau Small Area Income and Poverty Estimates school district file [80], data from the National Center for Education Statistics (NCES) [81], and the SFA Verification Summary Report 2012–2013 [82]. District racial/ethnic distribution and locale were based on data from NCES [83]. District race/ethnicity was coded as ≥50% white, ≥50% black, ≥50% Hispanic, and other. Region was based on Census region classifications [84] of each state.

### 2.3. Analysis Methods

Of the 518 SFAs with completed SFA Director Surveys, four were missing data on one or more control variables or on both the LWP comprehensiveness and SFA wellness policy implementation outcome variables and were excluded from all analyses. This left 514 SFAs in the analytical sample, located in 47 states and DC, only excluding Alaska, Hawaii, and Rhode Island. Across the 47 states and DC, there were a mean of 10.71 SFAs per state in the analytical sample (SD: 10.60), with a range of 1–49 SFAs per state (25th percentile: 4, 75th percentile: 14.5). Samples ranged from 493–514 SFAs for specific analyses due to item-specific missing data.

To align with our study questions, we used a combination of structural equation models (SEM) and generalized linear models (GLM) to test our study hypotheses. First, a mediation analysis was conducted with a SEM. SEM was used instead of other mediation approaches in order to properly account for the ordinal mediator, i.e., LWP comprehensiveness, which was a tertiled measure [85]. This model linked state wellness policy requirement laws to district LWP comprehensiveness, and state wellness policy requirement laws and district LWP comprehensiveness to SFA wellness policy full implementation, controlling for SFA and district characteristics. The SEM treated the tertiled district LWP comprehensiveness measure as an ordinal variable with a probit link, which imposed the parallel lines assumption [86]. The SEM treated the SFA implementation scale as a continuous measure, because it was not possible to fit a fractional logit model in the context of the SEM using Mplus. As a sensitivity check, we fit a generalized ordered probit model for this outcome (outside of the full mediation analysis) and tested whether coefficients were the same for both levels of district LWP comprehensiveness; the resulting F statistic was not statistically significant, supporting the parallel lines assumption.

Second, a GLM from the binomial family with logit link was run to link the SFA wellness policy full implementation scale (scaled as a proportion ranging from 0–1) to the state wellness policy requirement law indicator, controlling for SFA and district characteristics. This GLM allowed us to estimate a fractional logit model that properly accounts for the limited range of the SFA implementation scale. The adjusted mean percentage of items that were fully implemented by the SFA with versus without a state wellness policy requirement law was computed based on predicted values from this model. This model was generated as a sensitivity analysis to determine whether the association between state wellness policy requirement laws and SFA implementation holds up when accounting for the limited range of the SFA implementation measure.

All analyses accounted for the survey design and weights. Descriptive statistics and the GLM were computed in Stata/SE (version 15.1, StataCorp LP, College Station, TX, USA; 2016), while the SEM was computed in Mplus (version 8, Muthén & Muthén, Los Angeles, CA, USA; 2017). The sensitivity check of the parallel lines assumption was conducted in Stata/SE 15.1 using the *gologit2* user-written command [86,87].

## 3. Results

### 3.1. Sample Characteristics

Table 1 presents survey-weighted characteristics of the analytical sample overall and for SFAs located in states with wellness policy requirement laws versus those located in states without state wellness policy requirement laws. On average, SFAs fully implemented slightly less than half (5.28) of the 11 possible items in our SFA wellness policy full implementation scale. District LWP comprehensiveness tertiles were slightly unbalanced due to clustering in the continuous comprehensiveness measure. Districts in the lowest tertile of comprehensiveness addressed zero to just under half of the items in our scale, while those in the highest tertile addressed about two-thirds to 91% of the 12 items measured. The mean continuous district LWP comprehensiveness was 53.30 (SD: 21.01; not shown in tables), indicating that just over half of the items were addressed on average.

Over three-quarters of SFAs (76.03%) were in a district with ≥50% white students. About two-fifths of SFAs (41.01%) had a district child poverty rate of 20% or higher; the mean district child poverty rate was 19.20% (SD: 9.65%; not shown in tables). Nearly half of SFAs were in a rural locale (45.19%) and had fewer than 1000 students (49.57%), while only 13.54% were in a large to mid-size city and 13.32% had more than 5000 students. SFAs were located in all Census regions.

Nearly one-quarter of SFAs (22.97%) were in a state (*n* = 14 states and the District of Columbia, D.C.) with a state wellness policy requirement law; the remaining SFAs (77.03%) were in a state without a state wellness policy requirement law. The state wellness policy requirement laws were codified in both statutes (7 states and the District of Columbia) and administrative regulations (7 states) and were effective between 2004 (concurrent with the passing of the federal law instituting the original LWP mandate) and 2014. The only statistically significant bivariate differences between SFAs in states with versus without wellness policy requirement laws were for the SFA wellness policy full implementation scale (*p* = 0.013), SFA size (*p* = 0.002), and region (*p* < 0.001).

### 3.2. Relationship between State Law, District LWP Comprehensiveness, and SFA LWP Implementation

The results from the SEM mediation analysis linking state wellness policy requirement laws to district LWP comprehensiveness to SFA wellness policy full implementation are summarized in Table 2. State wellness policy requirement laws were positively associated with district LWP comprehensiveness (coeff.: 0.463; 95% CI: 0.123, 0.803) and SFA implementation (coeff.: 1.392; 95% CI: 0.299, 2.485), while district LWP comprehensiveness was also positively associated with SFA implementation (coeff.: 0.562; 95% CI: 0.072, 1.053). The indirect association between state wellness policy requirement laws and SFA wellness policy full implementation through district LWP comprehensiveness was not significant, suggesting that district LWP comprehensiveness did not mediate the relationship between state wellness policy requirement laws and SFA wellness policy full implementation. Finally, SFAs in the Northeast compared to the West had lower district LWP comprehensiveness and SFAs with <1000 students had higher SFA implementation compared to those with >5000 students. We compared these results to results from unadjusted bivariate models and results were similar for all three primary associations between state wellness policy requirement laws and district LWP comprehensiveness (ordered probit regression coeff.: 0.37; 95% CI: 0.04, 0.71; non-survey-adjusted Spearman’s rank correlation: 0.08) and SFA implementation (linear regression coeff.: 1.52; 95% CI: 0.32, 2.72; non-survey-adjusted Spearman’s rank correlation: 0.09); and between district LWP comprehensiveness and SFA implementation (tertile 2 versus tertile 1 linear regression coeff.: −0.02; 95% CI: −1.01, 0.96; tertile 3 versus tertile 1 linear regression coeff.: 1.76; 95% CI: 0.40, 3.11; non-survey-adjusted Spearman’s rank correlation: 0.05).

**Figure 1:**
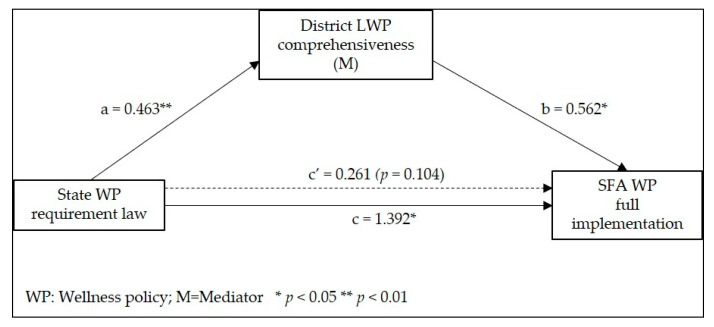
Mediation analysis path diagram.

Figure 1 illustrates the mediation analysis path diagram. Path a shows the association of state wellness policy requirement law with district LWP comprehensiveness; path *b* shows the association between district LWP comprehensiveness and SFA wellness policy full implementation; path *c* shows the direct association between state wellness policy requirement laws and SFA full wellness policy implementation; and path *c’* shows the indirect association of state wellness policy requirement laws with SFA full wellness policy implementation through district LWP comprehensiveness.

The mediation analysis path diagram graphically depicts the results presented in Table 2 and further illustrates that state wellness policy requirement laws were significantly associated with district LWP comprehensiveness (*a* path) and that district LWP comprehensiveness was significantly associated with SFA wellness policy full implementation (*b* path). There was a significant direct association between state wellness policy requirement laws and SFA implementation (*c* path), but no indirect association through district LWP comprehensiveness, as demonstrated by the insignificant *c’* path.

Results from the GLM sensitivity analysis linking state wellness policy requirement laws to SFA wellness policy full implementation are presented in Table 3. As noted with Table 2 and Figure 1, state wellness policy requirement laws were positively associated with SFA implementation (coeff.: 0.62, 95% CI: 0.18, 1.07). This equates to SFAs reporting full implementation of 59.56% of items in the scale in states with wellness policy requirement laws as compared to only 44.57% of the items without the state law requirement. As noted above in the SEM model, having a smaller SFA enrollment (fewer than 1000 students) as opposed to having more than 5000 students was also positively associated with SFA implementation.

## 4. Discussion

To our knowledge, this is the first study to examine the association between state wellness policy requirement laws, district LWP comprehensiveness, and SFA full wellness policy implementation. Consistent with our hypothesis, state wellness policy requirement laws were associated with district LWP comprehensiveness, and district LWP comprehensiveness was associated with SFA full wellness policy implementation. We discuss the state law-related findings further below. It is encouraging that district LWP comprehensiveness was associated with implementation; it supports the notion that having a formal, written policy does help to provide a framework for implementation and runs counter to arguments and/or findings that LWPs are not associated with school practices [30,32,33,51]. One possible explanation for the contrary findings between the prior work and this study is that the prior work was conducted within single states (as opposed to nationwide), focused on specific elements (e.g., physical activity, nutrition) of wellness policies (not overall wellness policy content), and/or conducted within a few years of the wellness policy mandate [30,31,32,51,58]. Our study was conducted nationwide, 9 years after the LWP mandate first took effect, and focused on overall policy content and implementation practices (rather than specific elements of the LWPs). In fact, our findings are consistent with prior work showing an association between LWPs overall and implementation practices, suggesting that the overall wellness policy may be more important for implementation rather than individual policy components [34,46]. Additionally, a strength of this study was that we were able to match the LWP components directly to the SFA wellness policy implementation components. Prior research likely did not have this level of precision and this is an area for continued study.

We also hypothesized that the district LWP comprehensiveness would mediate the relationship between state wellness policy requirement laws and SFA wellness policy full implementation. We did not find mediation. One possible explanation for this is that the mediator only measured the extent to which district LWP “addressed” (comprehensiveness) rather than “required” (strength) specific provisions. Prior research has clearly documented that most wellness policies in the United States are weak; in other words, they are comprehensive (addressing many topics), but they are not definitively required (strong) [6,9,22]. Additionally, implementation has been greater in districts with stronger and more comprehensive wellness policies overall [34,47,52]. Due to the low prevalence of strong district LWP overall in our sample, it was not possible to model LWP strength; future research should explore this issue further. Additionally, given the expanded oversight by state agencies regarding LWP, this is an area for states to work on with districts as they review LWP progress as part of the required triennial reviews that they will be conducting [3,4].

One of the unique aspects of this study was its focus on state wellness policy requirement laws, which include state laws requiring that districts submit their LWP to the state for review and/or compliance checks. To our knowledge, no other study has examined or reported on the prevalence of these laws. Given the association between state wellness policy requirement laws and both district LWP comprehensiveness and SFA wellness policy full implementation, this finding is noteworthy. Most advocacy efforts in the United States have focused specifically on strengthening LWPs at the district level. However, our findings suggest that there is also an opportunity for state legislatures and state agencies responsible for oversight of these policies to adopt and implement state wellness policy requirements through statute and/or regulation. From an advocacy perspective, it is much easier for advocacy organizations to work through 50 state legislatures and their executive branch agencies than tens of thousands of school districts nationwide. The data from this study were laws in effect as of school year 2014–2015; at that time, 15 states and the District of Columbia had relevant laws in place. Currently, as of school year 2019–2020, 16 states and the District of Columbia have relevant laws (unpublished data compiled by the study authors). Thus, there is a clear opportunity for the remaining 34 states to act in this area, and such laws can provide a framework to support the LWP review that state agencies are required to conduct every three years [3,4].

Finally, although the state wellness policy requirement laws and district LWP comprehensiveness were both associated with SFA wellness policy full implementation, it is noteworthy that SFAs only reported fully implementing 5.28 out of 11 LWP-related items measured in SNMCS, on average. While it is encouraging that SFAs reported implementation for many of the items examined, it is still important to recognize that LWP implementation is not universal. This has been a consistent finding in the literature [32,51] and continues to be a challenge in this nationwide sample. Qualitative research has identified factors that have served as barriers to LWP implementation, including lack of resources to support implementation, lack of a designated lead for implementation, competing priorities, and lack of accountability [17,25,26,27,29,30,88].

### Study Limitations

The findings from this study should be considered within the context of the following limitations. First, this was a cross-sectional study; as such, all findings reported herein should be considered as correlational, reflecting associations, rather than causal. Second, the study was limited to only one school year (2014–2015) due to the single year of outcome data available from SNMCS. Were data to become available, future studies should examine the impact of state wellness policy requirement laws and district LWPs on LWP implementation over time. Third, our measure of implementation was reported by the SFA director. It is possible that if the SNMCS had surveyed other district-level officials, the reports of implementation might have varied. Understanding the role that SFA directors play versus other district-level officials (e.g., district wellness policy champion, district wellness policy committee) in overseeing LWP implementation is an area for future study. Given that the federal LWP final rule required that all districts identify a key person responsible for LWP implementation [3], it will be helpful to document who the responsible officials are prospectively to guide future studies of LWP implementation. Relatedly, the LWP implementation measure was self-reported by the SFAs, which is subject to respondent bias and recall and may not fully capture actual implementation had it been directly observed. However, given the scope of what was captured in the LWP implementation measures in SNMCS, it would be difficult to objectively measure these items in their entirety, and it would require some level of reporting by an SFA or other district authority in addition to any specific objective measures.

## 5. Conclusions

In conclusion, although there has been a vast amount of research studying LWP development, content, and implementation over the past decade and a half, this study uniquely contributes to the LWP literature. Specifically, this study adds evidence regarding the role that both state laws and district policies can play in supporting LWP implementation. Yet, clearly more work is needed to bolster LWP implementation nationwide. Findings from this study and prior research suggest that state laws, combined with strong district LWPs, implementation support, and district and school stakeholder buy-in, collectively, will be needed to advance LWP implementation efforts, given that implementation is still far from universal.

## Figures and Tables

**Table 1:** School food authority survey—weighted sample characteristics.

Variable	Overall SFA Sample	SFA Sample in States with WP Requirement Laws	SFA Sample in States without WP Requirement Laws
	% or Mean (95% CI or SD)		
SFA Wellness Policy Full Implementation Scale (0–11) (Mean)	5.28 (SD: 3.87)	6.45 (SD: 3.99)	4.93 (SD: 3.77)
District LWP Comprehensiveness			
Tertile 1 (0.00–46.97)	37.79 (31.76, 44.23)	24.89 (16.90, 35.06)	41.75 (34.30, 49.60)
Tertile 2 (>46.97–64.65)	36.35 (29.75, 43.51)	42.05 (29.00, 56.31)	34.61 (27.20, 42.85)
Tertile 3 (>64.65–91.29)	25.85 (20.34, 32.25)	33.07 (20.33, 48.88)	23.64 (17.89, 30.55)
State Wellness Policy Requirement Law	22.97 (18.06, 28.75)		
District Race/Ethnicity			
≥50% White	76.03 (70.91, 80.51)	81.31 (69.05, 89.45)	74.46 (68.71, 79.47)
≥50% Black	7.04 (4.34, 11.23)	7.94 (2.58, 21.95)	6.77 (4.02, 11.17)
≥50% Hispanic	8.82 (6.27, 12.28)	3.49 (1.28, 9.14)	10.41 (7.27, 14.69)
Other	8.11 (5.74, 11.34)	7.27 (3.65, 13.96)	8.36 (5.61, 12.28)
District Child Poverty Rate			
<20%	58.99 (53.38, 64.37)	63.25 (50.26, 74.57)	57.72 (51.54, 63.66)
≥20%	41.01 (35.63, 46.62)	36.75 (25.43, 49.74)	42.28 (36.34, 48.46)
District Locale			
Large to mid-size city	13.54 (10.09, 17.93)	8.95 (3.57, 20.69)	14.91 (10.95, 19.97)
Suburban	20.50 (16.90, 24.64)	33.65 (23.58, 45.46)	16.58 (12.98, 20.93)
Rural	45.19 (38.78, 51.77)	36.59 (23.78, 51.63)	47.76 (40.38, 55.23)
Township	20.77 (15.49, 27.28)	20.81 (11.28, 35.20)	20.76 (14.74, 28.42)
SFA Size			
Fewer than 1000 students	49.57 (44.23, 54.92)	30.59 (19.21, 44.97)	55.23 (49.42, 60.90)
1000–5000 students	37.12 (31.92, 42.63)	53.65 (40.15, 66.64)	32.18 (26.87, 38.00)
More than 5000 students	13.32 (11.09, 15.91)	15.75 (10.48, 23.00)	12.59 (10.10, 15.58)
Region			
West	17.49 (12.92, 23.24)	12.63 (7.18, 21.28)	18.93 (13.39, 26.09)
Midwest	39.79 (33.29, 46.67)	16.97 (9.18, 29.26)	46.60 (39.10, 54.25)
South	24.79 (19.95, 30.36)	39.01 (26.45, 53.21)	20.55 (16.27, 25.60)
Northeast	17.94 (13.30, 23.75)	31.39 (21.08, 43.93)	13.92 (9.05, 20.82)

SFA, School Food Authority; LWP, Local Wellness Policy; WP, Wellness Policy; CI, Confidence Interval; SD, Standard Deviation. Overall sample *n* = 493–514 SFAs, due to item-specific missing data; sample in states with WP requirement laws *n* = 123–127 SFAs, due to item-specific missing data; sample in states without WP requirement laws *n* = 370–387, due to item-specific missing data.

**Table 2:** Structural equation model linking state wellness policy requirement laws, district LWP comprehensiveness, and SFA wellness policy full implementation.

Predictor or Control Variable	Outcome Variables	
	District LWP Comprehensiveness	SFA Wellness Policy Full Implementation
	Coefficient (95% CI)	Coefficient (95% CI)
State Wellness Policy Requirement Law	0.463 ** (0.123, 0.803)	1.392 * (0.299, 2.485)
District LWP Comprehensiveness		0.562 * (0.072, 1.053)
District Race/Ethnicity		
≥50% White	Referent	Referent
≥50% Black	0.426 (−0.109, 0.962)	−0.510 (−2.231, 1.210)
≥50% Hispanic	0.440 (−0.042, 0.923)	0.295 (−1.422, 2.011)
Other	−0.233 (−0.655, 0.189)	−0.088 (−1.569, 1.393)
District Child Poverty Rate		
<20%	Referent	Referent
≥20%	−0.089 (−0.428, 0.250)	−0.107 (−1.085, 0.871)
District Locale		
Large to mid-size city	Referent	Referent
Suburban	0.297 (−0.128, 0.722)	−1.142 (−2.827, 0.543)
Rural	0.388 (−0.062, 0.839)	−1.690 (−3.419, 0.039)
Township	0.346 (−0.194, 0.885)	−0.332 (−2.140, 1.477)
SFA Size		
Fewer than 1000 students	0.054 (−0.315, 0.424)	1.761 * (0.389, 3.132)
1000–5000 students	0.032 (−0.297, 0.360)	0.898 (−0.243, 2.038)
More than 5000 students	Referent	Referent
Region		
West	Referent	Referent
Midwest	−0.173 (−0.568, 0.222)	−0.755 (−2.357, 0.846)
South	−0.215 (−0.634, 0.204)	0.403 (−1.029, 1.835)
Northeast	−0.539 * (−1.014, −0.064)	−0.644 (−2.368, 1.081)
Intercept/ Thresholds	−0.078 (−0.576, 0.420), 0.921 *** (0.413, 1.428)	5.054 *** (3.125, 6.983)
Residual Variances		13.305 *** (9.331, 17.280)
Indirect association between state requiring district wellness policy and SFA wellness policy full implementation	0.261 (−0.053, 0.574)	

SFA, School Food Authority; LWP, Local Wellness Policy; CI, Confidence Interval. *n* = 514 SFAs. * *p* < 0.05, ** *p* < 0.01, *** *p* < 0.001.

**Table 3:** Association between state wellness policy requirement law and SFA wellness policy full implementation.

Variable	Coefficient (95% CI)
State Wellness Policy Requirement Law	0.62 ** (0.18, 1.07)
District Race/Ethnicity	
≥50% White	Referent
≥50% Black	−0.09 (−0.92, 0.74)
≥50% Hispanic	0.21 (−0.44, 0.86)
Other	−0.08 (−0.64, 0.47)
District Child Poverty Rate	
<20%	Referent
≥20%	−0.06 (−0.46, 0.34)
District Locale	
Large to mid-size city	Referent
Suburban	−0.37 (−0.92, 0.19)
Rural	−0.55 (−1.12, 0.02)
Township	−0.04 (−0.73, 0.65)
SFA Size	
Fewer than 1000 students	0.68 ** (0.19, 1.16)
1000–5000 students	0.35 (−0.10, 0.79)
More than 5000 students	Referent
Region	
West	Referent
Midwest	−0.32 (−0.92, 0.27)
South	0.11 (−0.47, 0.69)
Northeast	−0.36 (−0.93, 0.22)
Adjusted Mean	
With state wellness policy requirement	59.56%
Without state wellness policy requirement	44.57%

SFA, School Food Authority. *n* = 509 SFAs, due to item-specific missing data. ** *p* < 0.01.

## Data Availability

Requests for access to the public use SNMCS data should be submitted via electronic mail to: FNSStudies@usda.gov.
